# Latent Profile Analysis of Generic Conspiracy Beliefs‐ Differences in Science Acceptance, Analytical and Intuitive Thinking

**DOI:** 10.1111/sjop.70120

**Published:** 2026-05-22

**Authors:** Peter Karlsson, Torbjörn Josefsson, Sara Svensson, Linus Andersson

**Affiliations:** ^1^ School of Health and Welfare Halmstad University Halmstad Sweden; ^2^ School of Education, Humanities and Social Sciences Halmstad University Halmstad Sweden

**Keywords:** analytic and intuitive thinking, conspiracy beliefs, latent profile analysis, science denial

## Abstract

The aims of the present study were to (i) identify profiles of belief in conspiracy theories, and to (ii) test whether the obtained profiles differed in analytical (rational ability, rational engagement, Cognitive Reflection Test) and intuitive thinking (experiential ability, experiential engagement), and science denial (vaccine, climate science). In total, 451 participants (Mean age = 30.80, SD = 16.59, 61% female) were recruited from high‐schools and social media groups. The participants completed questionnaires and performance tests of the study variables. The Latent Profile Analysis identified four distinct profiles that showed consistent and distinct response patterns over all five subscales of the Generic Conspiracy Belief Scale, with no overlap between the profiles, ranging from low (Profile 1) to strong conspiracy beliefs (Profile 4). Profile 1 was the largest class (38.14%), whilst Profile 4 was the smallest class (8.61%). The analyses further revealed that no differences were found between profiles in analytical thinking, measured by the performance‐based cognitive reflection test. However, profile differences were found for self‐reported analytical thinking, intuitive thinking, and science denial. Overall, less conspiracy beliefs were accompanied by less intuitive thinking and higher acceptance of climate and vaccine science. Somewhat unexpectedly, participants who held the strongest conspiracy beliefs (Profile 4) as well as those who had the lowest beliefs in conspiracy theories (Profile 1) reported higher levels of analytical thinking, compared with the other two profiles, suggesting that people who most strongly endorse conspiracy theories report high use of both analytical and intuitive thinking styles. A potential explanation may be that conspiracy beliefs initially originate through intuitive thinking, while analytical thinking can be used to maintain and justify these beliefs through, for instance, motivated reasoning.

## Key Points

- Latent Profile Analysis identified four distinct profiles of participants that showed consistent, and distinct, non‐overlapping response patterns over all five subscales of the Generic Conspiracy Belief Scale (GCBS).
- Less belief in conspiracy theories was associated with less self‐reported intuitive thinking, and higher acceptance of climate and vaccine science.
- Participants who held the strongest conspiracy beliefs (Profile 4) and those who held the lowest beliefs in conspiracy theories (Profile 1) reported higher levels of analytical thinking, compared with the other two profiles.

## 1 Introduction

Conspiracy theories are common on social media, the internet, and in social and political discourses (e.g., Douglas et al. 2019; Teovanović et al. 2021). Research indicates that belief in unfounded conspiracy theories can have serious consequences both for individuals and societies (Douglas et al. 2019). For instance, Teovanović et al. (2021) found that belief in conspiracy theories concerning COVID‐19 was related to reduced intention to follow recommended guidelines (e.g., social distance), increased engagement in pseudoscientific practices (e.g., consuming colloidal silver), and decreased intention to receive Covid‐19 vaccine. Furthermore, research indicates that belief in conspiracy theories is also related to increased prejudice (e.g., Jolley et al. 2020), violence, radicalization, and extremism (e.g., Bartlett and Miller 2010; Uscinski and Parent 2014), and diminished trust in government and institution (Douglas et al. 2019). Further, a growing body of research has also indicated that belief in conspiracy theories is related to science denial, that is rejecting or distrusting established scientific evidence or the scientific method as a means to gain reliable evidence (e.g., Lewandowsky, Gignac, et al. 2013; Lewandowsky et al. 2015; Uscinski et al. 2017). In light of the negative consequences belief in conspiracy theories can have, for both individuals and societies, it is imperative to gain a better understanding of how common (various degrees of) belief in conspiracy theories is, and what factors are related to differences between people more versus less susceptible to belief in conspiracy theories.

### 1.1 Conspiracy Theory and Conspiracy Mindset

Conspiracy theory has been defined as “an unverified and relatively implausible allegation of conspiracy, claiming that significant events are the result of a secret plot carried out by a preternaturally sinister and powerful group of people” (Brotherton and French 2014, 238). Douglas and Sutton (2023) propose a more comprehensive definition where conspiracy theory is seen as:

a belief that two or more actors have coordinated in secret to achieve an outcome, and that their conspiracy is of public interest, but not public knowledge. From these defining features of conspiracy theory, other characteristics follow logically. Thus, Conspiracy theories: (*a*) are oppositional, which means they oppose publicly accepted understandings of events; (*b*) describe malevolent or forbidden acts; (*c*) ascribe agency to individuals and groups rather than impersonal or systemic forces; (*d*) are epistemically risky, meaning that though individual conspiracy theories are not necessarily false or implausible, conspiracy theories taken collectively are more prone to falsity than other types of belief; and (*e*) are social constructs that are not merely adopted by individuals but shared with social objectives in mind, and that have the potential not only to represent and interpret reality but also to fashion new social realities. (287)

Moreover, several researchers (e. g., Frenken and Imhoff 2021; Imhoff et al. 2022; Uscinski et al. 2017) have highlighted the need to distinguish beliefs in specific conspiracy theories from a general conspiracy mindset, also labeled as conspiracy mentality (Frenken and Imhoff 2021; Imhoff et al. 2022), described as “a general tendency to believe in conspiracy theories” (Frenken and Imhoff 2021, 1–2), that basically reflects an enduring and fundamental conspiratorial view of the world. Hence, an individual with a strong conspiratorial worldview may be more prone to explain significant events as a product of a conspiracy than a less conspiracy minded person. However, even if individuals may have a predisposition towards conspiratorial thinking, it does not necessarily mean that they embrace every single conspiracy theory. Thus, it is possible to have a general conspiracy mindset but neglect certain conspiracy theories (Uscinski et al. 2017). Nevertheless, numerous correlational studies show that people who believe in one conspiracy theory also tend to believe in several other conspiracy theories (e.g., Douglas and Sutton 2018; Lewandowsky, Oberauer, et al. 2013).

Studies using Latent Profile Analysis (LPA) have reported results that are consistent with the notion that belief in conspiracy theories represent a general mindset, both regarding a wide range of different conspiracy theories (Frenken and Imhoff 2021) and Covid‐19 conspiracy theories specifically (Agley and Xiao 2021; Jones et al. 2023). Overall, the latent profiles identified in these studies differed in degree of conspiracy beliefs but were largely non‐overlapping, indicating a rather high degree of consistency in conspiracy beliefs over different conspiracy theories (i.e., participants reporting a high degree of belief in one conspiracy theory tend to report high belief in other conspiracy theories). Further, the profiles reporting the highest degree of conspiracy beliefs tended to be the smallest profiles identified, with the larger profiles reporting low to moderate conspiracy beliefs (Agley and Xiao 2021; Frenken and Imhoff 2021; Jones et al. 2023).

### 1.2 Analytical and Intuitive Thinking

In accordance with dual‐process theories, cognitive processes can be characterized by two distinct types of information processing. One intuitive form of processing, often referred to as Type 1 thinking, or processes, that is more automatic, fast, effortless, and one analytical form of processing called Type 2 thinking/processes that is more resource demanding, analytical, slower, and more deliberate (see for instance Evans 2008; Evans and Stanovich 2013; Pennycook et al. 2015a). A large body of research indicates that intuitive thinking (positive correlation) and analytical thinking (negative correlation) are related to epistemically suspect beliefs (i.e., beliefs not supported by modern science), such as paranormal beliefs (e.g., Pennycook et al. 2012; Šrol 2022; Svedholm and Lindeman 2013), religious belief (e.g., Pennycook et al. 2012), pseudoscientific beliefs (e.g., Šrol 2022), and conspiracy theories (e.g., Binnendyk and Pennycook 2022; Šrol 2022; van Prooijen 2017). A recent meta‐analysis concluded that both self‐report measures and performance‐based measures of analytical thinking are negatively correlated with both generic and specific conspiracy beliefs (with about medium effect sizes; Yelbuz et al. 2022).

One performance‐based measure of analytical thinking often used in research concerning belief in conspiracy theories is the Cognitive Reflection Test (CRT) (Frederick 2005). The CRT consists of problems that induce an intuitive, but incorrect, answer. To answer these problems correctly, participants need to engage in more analytical, type 2 thinking (Binnendyk and Pennycook 2022; Frederick 2005; Pennycook et al. 2015a).

Several previous studies have reported negative correlations between performance on the CRT and belief in specific conspiracy theories (Alper et al. 2021; Jedinger et al. 2023; Ozono and Sakakibara 2024; Stanley et al. 2021) as well as generic conspiracy theories (e.g., Alper et al. 2021; Ozono and Sakakibara 2024). However, other studies find that the relationship depends on moderators, such as epistemic rationality (with a relationship only for participants valuing epistemic rationality; Ståhl and Van Prooijen 2018) or types of conspiracy belief measured (with a relationship only regarding conspiracy theories judged as implausible in a pilot‐study, not plausible ones; Hattersley et al. 2022). Finally, Rizeq et al. (2021) reported a non‐significant association between performance on the CRT and generic conspiracy beliefs (GCBS) when cognitive ability, Actively Open Thinking, and Ontological Confusion were included in the same model, only ontological confusion predicted belief in conspiracy theories. In all, these results indicate that the association between performance on the CRT and conspiracy beliefs is rather complex and is moderated by both aspects of the conspiracy theories studied (i.e., plausibility), motivational factors (i.e., valuing epistemic rationality), and dependent on other factors included in the analyzed models.

Previous studies have also used self‐report measures concerning analytical and intuitive thinking. In their systematic review, Van Mulukom et al. (2022) concluded that higher degrees of self‐reported intuitive thinking, and less analytical thinking, were related to more belief in covid‐19 conspiracy theories. Yelbuz et al. (2022) reported, in their meta‐analysis, that self‐reported analytical (reflective) thinking was negatively related to belief in both specific and generic conspiracy theories. Oftentimes, these studies have utilized some version of the Rational‐Experiential Inventory (REI) (see for instance Pacini and Epstein 1999). The REI measure is based on dual‐processing models and measures intuitive, or experiential (intuitive, fast, and effortless, in accordance with Type 1 thinking) and analytical, or rational (analytical, slow and effortful, i.e., Type 2) thinking styles, and includes two subscales concerning self‐reported engagement (or preference) and ability for each style of thinking (Pacini and Epstein 1999). The REI has been translated into several languages and comes in both longer and shorter versions (e.g., Björklund and Bäckström 2008; Georgiou et al. 2021; Pacini and Epstein 1999).

Results concerning the association between self‐reported intuitive and analytical thinking are somewhat mixed. Several previous studies, using various forms of REI, have reported significant positive associations between self‐reported intuitive thinking and belief in specific conspiracy theories (e.g., Georgiou et al. 2019; Pytlik et al. 2020; Swami et al. 2014), as well as generic conspiracy theories (e.g., Georgiou et al. 2019). Likewise, several studies have also reported significant negative associations between self‐reported analytical thinking and belief in specific conspiracy theories (e.g., Georgiou et al. 2019; Gligorić et al. 2021; Ozono and Sakakibara 2024; Swami and Barron 2021) as well as generic conspiracy theories (e.g., Georgiou et al. 2019; Gligorić et al. 2021; Jastrzębski and Chuderski 2022; Ozono and Sakakibara 2024). However, some studies report non‐significant associations between belief in conspiracy theories and intuitive thinking (Gligorić et al. 2021), and analytical thinking (Pytlik et al. 2020; Georgiou et al. 2021). Finally, Jones et al. (2023) investigated differences between the three identified profiles concerning belief in Covid‐19 conspiracy theories in self‐reported intuitive and analytical thinking measured using the REI. The profile with lowest belief in Covid‐19 conspiracy theories reported less reliance on intuitive thinking compared to the profiles with higher belief, but Jones et al. (2023) found evidence of a non‐linear association between degree of conspiracy beliefs and self‐reported analytical thinking as the profiles reporting the lowest and highest conspiracy beliefs reported more reliance on analytical thinking compared to the profile with moderate belief.

### 1.3 Science Denial

Science denialism has been defined as “the dismissal of well‐established scientific evidence or the scientific method as a means to gather reliable evidence” (Jylhä et al. 2022, 151) and has been seen as an expression of pseudoscientific views that are characterized by scientific statements that suffer heavily from a lack of reliability but still claim to be the most, if not the only, reliable knowledge available on the subject at hand (Hansson 2013, 2017). Jylhä and colleagues (Jylhä et al. 2022) emphasize that science denial should be distinguished from skepticism because a healthy dose of skepticism when confronted with statements and conclusions based on novel (and questionable) evidence is an important aspect of what characterizes science.

Several authors have also identified the frequent belief in, and use of, conspiracy theories as a key characteristic of science denial (Diethelm and McKee 2009; Douglas and Sutton 2015; Hansson 2017), with science denial being related to both belief in more general conspiracy theories (i.e., not related to science or scientists) and belief in conspiracy theories directly related to science and/or scientists (Rutjens and Većkalov 2022).

In general, it is a common belief among science denialists that the entire population of scientists in a particular area are engaged in a conspiracy, designed to cover up the truth (Diethelm and McKee 2009; Washington and Cook 2011). For example, climate change denialists frequently state that global warming is just a hoax, and that empirical data have been fabricated by climate scientists in order to receive research funding (Douglas and Sutton 2015). A recent meta‐analysis found moderate to large negative correlations between beliefs in climate change conspiracy theories and acceptance of climate science, and acceptance of science in general (Biddlestone et al. 2022). For example, Lewandowsky, Gignac, et al. (2013) reported significant negative correlations between belief in specific science related conspiracy theories (regarding climate change, vaccines, and GM food) and acceptance of the corresponding science (average acceptance of scientific propositions regarding climate change, vaccine, and GM food respectively). Furthermore, they also reported that general conspiracist ideation was significantly, and negatively, related to acceptance of climate, vaccine, and GM food science. In a similar study, Lewandowsky, Oberauer, et al. (2013) reported that general (i.e., non‐science related) conspiracist ideation was negatively related to acceptance of both climate science and acceptance of other sciences (e.g., HIV‐AIDS, smoking and lung cancer). In a recent study, Marques et al. (2021) reported small to moderate associations between the five GCBS subscales and anti‐science attitudes towards several topics (e.g., GM food, 5G, childhood vaccinations, nuclear power, wind power, solar power, and recycled water).

Agley and Xiao (2021) reported that trust in science was a strong and significant predictor of profile membership in their study, with lower trust in science associated with a higher probability of belonging to either of the three profiles showing higher degrees of belief in conspiracy theories compared to the profile showing the lowest belief in conspiracy theories concerning Covid‐19.

### 1.4 The Present Study

A large number of previous studies have established that cognitive thinking styles and abilities, as well as science denial, are correlated with belief in conspiracy theories (e.g., Biddlestone et al. 2022; Yelbuz et al. 2022). As recommended by van Prooijen and Imhoff (2022), we made an effort to extend previous correlational research by adopting a person‐oriented approach. In the present study, we used LPA to investigate profiles of beliefs in conspiracy theories. As opposed to variable‐centered approaches, where all participants are treated as a single group, LPA makes it possible to identify distinct profiles within a sample where each profile shares a common response pattern that differs in a meaningful and interpretable way from the other profiles. Hence, a person‐centered approach may provide an important contribution to the current field of research because the LPA analysis can reveal a more nuanced and fine‐grained understanding of belief in conspiracy theories compared with more traditional variable‐centered approaches. We further explore how level of conspiracy belief interacts with cognitive thinking styles and science denial by investigating differences in these auxiliary variables between the identified latent profiles (Asparouhov and Muthén 2014). By using the BCH method to explore differences between the identified profiles, we are able to find potential non‐linear associations between conspiracy beliefs and auxiliary variables.

Thus, the aims of the present study are to (i) examine the robustness of previous findings of general conspiracy belief profiles, using LPA to identify profiles of belief in conspiracy theories (Frenken and Imhoff 2021), (ii) explore differences between identified profiles in cognitive thinking styles and science denial (climate science and vaccine science).

## 2 Methods

### 2.1 Participants

In total, 451 participants (Mean age = 30.80, SD = 16.59, 61% female) were recruited from two Swedish populations (a general adult population from 18 years and older, and a population consisting of high school students). The adult sample (*N* = 237, Mean age = 43.70, age range 18–82, SD = 13.20, 63% female) was based on data from an online survey. In this sample, 66% of the participants reported university degree as their highest educational degree. The other sample consisted of high school students from eight different classes, recruited from three high schools (*N* = 214, Mean age = 16.58, SD = 0.66, age range 16–17, 59% female).

In all 21 participants were missing data on the Generic Conspiracy Beliefs Scale (four from the adult sample, and 17 from the high school sample) so the effective sample size for the analyses was 430 participants.

### 2.2 Measures

#### 2.2.1 Generic Conspiracy Beliefs Scale (GCBS)

The GCBS (Brotherton et al. 2013) is a 15‐item questionnaire designed to assess generic conspiracist ideation and consists of five subscales (Belief in routine governmental malfeasance, GM; Belief in the existence of malevolent global conspiracies, MG; Belief in the existence and cover‐up of extraterrestrials, ET; Concerns over the unethical control of information, CI; and Belief in conspiracies infringing on personal well‐being and liberty, PW). Responses were given on a 5‐point Likert scale, ranging from 1 (*definitively not true*) to 5 (*definitively true*). The instruction to the instrument is as follows: There is often debate about whether or not the public is told the whole truth about various important issues. This brief survey is designed to assess your beliefs about some of these subjects. Please indicate the degree to which you believe each statement is likely to be true on the following scale: Definitely not true; Probably not true; Not sure/cannot decide; Probably true; Definitely true.

The Swedish translation of the GCBS were done by the authors of the present study. An independent translator, blind to the original questionnaire, performed a back translation that resulted in minor changes on a few items. The initial translated version was pilot‐tested in a Swedish high‐school class, and the items were found to be understood and interpreted as originally intended. The original version of the GCBS has shown good psychometric properties (e.g., Brotherton et al. 2013; Drinkwater et al. 2020). See Tables S1 and S2 for all items and instructions for the original version and Swedish translation of the GCBS respectively.

#### 2.2.2 Rational‐Experiential Inventory (REI‐40)

The REI‐40 (Pacini and Epstein 1999) is a 40‐item scale that is designed to assess intuitive, or *experiential* (intuitive, fast, and effortless) and analytical, or *rational* (analytical, slow and effortful) thinking styles, and contains two subscales for each style of thinking; Experiential Engagement measures reliance on, and enjoyment of, relying on intuition and feelings when making decisions (e.g., “I like to rely on my intuitive impressions”), Experiential Ability measures level of ability in intuitive impressions and feelings (e.g., “I believe in trusting my hunches”), Rational Engagement measures reliance on and enjoyment of analytical and logical thinking (e.g., “I prefer complex problems to simple problems”), and Rational Ability, finally, measures level of ability to think analytically and logically (e.g., “I have a logical mind”) (Pacini and Epstein 1999). Instructions to participants was taken from (Björklund and Bäckström 2008): “Please rate the following statements about your feelings, beliefs and behaviors using the scale below. Work rapidly.” Responses were made on a 5‐point Likert‐scale (from 1 = *completely false* to 5 = *completely true*). Scores for the four subscales was calculated by averaging the items for each subscale, with higher scores indicating higher self‐reported engagement and ability in intuitive and analytical thinking respectively.

We used the Swedish version of REI‐40 that has shown satisfactory psychometric properties (Björklund and Bäckström 2008). Cronbach's *α* indicates good internal consistency for all four subscales in the present study, ranging from *α* = 0.79 (Experiential ability) to *α* = 0.82 (for each of the subscales Experiential engagement, Rational ability, and Rational engagement).

#### 2.2.3 Cognitive Reflection Test (CRT)

The CRT is also based on dual‐processing models (Frederick 2005). It consists of problems designed to suggest intuitive, but incorrect, answers and measures participants' propensity to override these intuitive answers and engage in analytical thinking to reach the correct answer (Frederick 2005; Thomson and Oppenheimer 2016). The CRT items in Frederick (2005), Oldrati et al. (2016), Primi et al. (2016), Thomson and Oppenheimer (2016), and Toplak et al. (2014) were translated, as verbatim as possible, to Swedish by the authors. All items were then pilot tested in a high school class and evaluated in terms of familiarity with the items and level of difficulty to minimize the risk of ceiling and floor effects. Four items were selected due to being easy to interpret correctly by all participants in the pilot study, not being familiar to any participant, and for giving the most even spread in correct‐incorrect answers.

Two items were taken from Frederick (2005), one item each was taken from Primi et al. (2016) and Toplak et al. (2014). See Table S3 for the original CRT items used and the Swedish translation. Number of correct answers as well as number of intuitive answers were analyzed. Intuitive answers refer to the number of times participants gave the intuitive, but incorrect, answer suggested in the problems.

#### 2.2.4 Acceptance of Climate and Vaccination Science

We used two subscales from the study by Lewandowsky, Gignac, et al. (2013) measuring acceptance of climate and vaccination science respectively. These two particular subscales were selected because they reflect common topics, frequently debated in pseudoscientific contexts (e.g., Lewandowsky, Gignac, et al. 2013), and were commonly debated also in Sweden at the time of data collection. Both subscales contain five items each, on a 5‐point Likert‐scale (1 = *strongly disagree*, 5 = *strongly agree*); Acceptance of climate science (e.g., “I believe that the climate is always changing and what we are currently observing is just natural fluctuation”), and Acceptance of vaccination science (e.g., “I believe that vaccines are a safe and reliable way to help avert the spread of preventable diseases”). Scores for the two science acceptance scales was calculated by averaging the items for each scale, with higher scores indicating higher acceptance of climate and vaccine science respectively.

For this study, the items were translated into Swedish using the forward‐backward translation technique, which ensures that the meaning and intent of the items are accurately preserved in both languages. The initial translated version was pilot‐tested in a Swedish high‐school class, and the items were found to be understood and interpreted as originally intended. See Tables S4 and S5 respectively for all items from the original version and the Swedish translation. Reliability estimates, Cronbach's *α*, indicate good internal consistency for both subscales in our study: Acceptance of climate science (*α* = 0.75), Acceptance of vaccination science (*α* = 0.91).

### 2.3 Procedure

Online survey participants were recruited through social media. We reached out to administrators of groups on social media (e.g., Facebook) and asked for permission to post information about our study and links to the questionnaire. In an effort to recruit participants that potentially would be vulnerable to conspiratorial thinking, we targeted social media groups whose profiles reflected an interest in subjects that previously have been associated with belief in conspiracy theories (e.g., alternative medicine/health, climate skepticism, anti‐vaccination, radiation, and a diverse range of groups with an interest in new‐age related topics, such as spiritual development). In addition, we also recruited participants from a variety of so called “general” social media groups (e.g., groups for university students, inhabitants in certain cities in Sweden, large corporations) that were not particularly targeted towards topics related to “alternative views”. The data were collected during September 1st through November 30th, 2022. All online participants first received written information about the study after clicking on the link to the study and then had to confirm that they consented to participate before they were taken to the next page (the first section) of the questionnaire.

For the high school sample, letters including a presentation of the current study and an inquiry to let their pupils participate were sent by email to five high schools in southwest Sweden, of which three schools agreed to take part in the study. The data were collected between December 9th, 2022, and November 17th, 2023. All measurements in the high school sample were conducted in classrooms at each high school.

The information given to all participants was based on the instructions in the original version of the General Conspiracy Beliefs Scale (Brotherton et al. 2013) and the REI (Björklund and Bäckström 2008), and specifically avoided using terms such as “conspiracy theory”, “conspiracy belief”, and similar terms (that may have a pejorative meaning to some participants) in order not to increase the risk of demand characteristics, social desirability, and similar biases in responding. The following information concerning the purpose of the study was given: “There is often debate about whether or not the public is told the whole truth about various important issues. This survey is designed to assess your beliefs about some of these subjects. The survey also includes questions concerning your feelings, beliefs and behaviors in relation to thinking, decision making and problem solving. The purpose of this study is to increase our knowledge regarding how people think about important issues.”

The APA ethical standards were followed in the conduct of the study. All participants were given written information about the study, see above (and the high school sample also received verbal information). Written informed consent was obtained from all participants included in the study, prior to the first data collection. Participants did not receive any compensation for taking part in the study. All procedures performed in studies involving human participants were in accordance with the ethical standards of the institutional and/or national research committee and with the 1964 Helsinki declaration and its later amendments or comparable ethical standards (World Medical Association 2024). The present study received ethical approval from the Swedish Ethical Review Authority (Dnr 2022–02402‐01).

### 2.4 Statistical Analysis

In the first step of the analysis we conducted a maximum likelihood confirmatory factor analysis, using MPlus version 8, to evaluate the factor structure of our Swedish translation of the GCBS. We used the following model fit indices to evaluate model fit: the *χ* ^2^ index of fit, comparative fit index (CFI), Tucker‐Lewis index (TLI), root mean square error of approximation (RMSEA), and standardized root mean square residual (SRMR). We used the following guidelines as indicating good model fit: CFI ≥ 0.95, TLI ≥ 0.95, RMSEA ≤ 0.06, and SRMR ≤ 0.06 (Brown 2015; Nye 2023).

In the next step, Latent Profile Analysis (LPA) was conducted using MPlus version 8 (Muthén and Muthén 2017) to identify latent profiles of conspiracy beliefs patterns. We used Maximum Likelihood and a robust estimator (MLR). LPA uses a person‐centered approach to identify unobserved groups of participants that have different patterns of conspiracy beliefs. We estimated models with an increasing number of latent profiles to determine the optimal solution (Agley and Xiao 2021).

To determine the model with the optimal number of profiles we considered statistical model fit indices, entropy (with values > 0.80 indicating good classification of participants into latent profiles), conceptual meaning, and the smallest estimated class proportions. The Model fit indices used were the Akaike Information Criterion (AIC), Bayesian Information Criterion (BIC), and Sample‐size adjusted BIC (SABIC), with smaller values representing greater model fit (Nylund‐Gibson and Choi 2018).

We further used the Vuong‐Lo–Mendell–Rubin adjusted likelihood ratio test (VLMR‐LRT) to assess if adding a profile leads to a significant improvement in model fit. The VLMR‐LRT compares two neighboring profile models. A *p*‐value of > 0.05 indicates that a model with *k* − 1 profiles has a better fit than a model with *k* profiles (Nylund‐Gibson and Choi 2018).

Lastly, we considered the conceptual meaning of identified profiles and smallest estimated class proportions to inform the selection of the model with the optimal number of profiles (Ferguson et al. 2020). Conceptual meaning refers to the interpretability of the identified profiles. The selected model must consist of profiles that represent distinct, meaningful, and interpretable groupings of participants. Concerning smallest estimated class proportions, we excluded models with profiles consisting of < 5% of the sample; this in order to avoid including spurious profiles and capitalizing on chance or common error variance (Ferguson et al. 2020).

We further conducted analyses of differences in auxiliary variables, between the identified latent profiles, using the BCH method (Asparouhov and Muthén 2014; Ferguson et al. 2020). We included the following auxiliary variables: Experiential Ability, Experiential Favorability, Rational Ability, Rational Favorability, Acceptance of Climate science, Acceptance of Vaccination science, CRT correct and CRT Intuitive. In addition, Age was included as an auxiliary variable to descriptively characterize the identified latent profiles. The inclusion of age was motivated by both theoretical and methodological considerations. From a methodological perspective, the present sample consisted of two partially distinct recruitment groups (high school students and adults), resulting in substantial age variation both between and within subsamples. Unlike education level, which is structurally tied to the sampling frame (i.e., distinguishing high school students from adult participants), age varies continuously within the adult sample and therefore allows for a more fine‐grained characterization of individual differences (Enders et al. 2024). From a theoretical perspective, age is commonly included as a sociodemographic variable in research on conspiracy beliefs and has shown modest negative associations with conspiratorial thinking in prior large‐scale studies (e.g., Bordeleau and Stockemer 2024; Douglas et al. 2019; Enders et al. 2024). Importantly, age was included as an auxiliary variable to aid interpretation and provide a descriptive characterization of the identified profiles (Asparouhov and Muthén 2014). In contrast, education level was not included due to its strong confounding with sampling group (high school vs. adult participants) (van Prooijen 2017). Furthermore, education is often interpreted as a proxy for underlying cognitive and epistemic variables, such as analytical thinking and scientific literacy, which are directly assessed in the present study (van Prooijen 2017; Yelbuz et al. 2022). Gender was not included as an auxiliary variable given that prior research shows inconsistent, generally weak, and context‐dependent associations between gender and conspiracy beliefs (e.g., Cassese et al. 2020; Douglas et al. 2019). Consequently, as the present study focuses on cognitive and epistemic variables, gender was not considered theoretically central to the research aims.

## 3 Results

### 3.1 Confirmatory Factor Analysis

First, we conducted a maximum likelihood confirmatory factor analysis, using MPlus version 8, to evaluate our Swedish translation of the GCBS. The confirmatory factor analysis indicated good model fit, *χ* ^2^(80) = 199.96, *p* < 0.001, CFI = 0.977, TLI = 0.970, RMSEA = 0.059 (90% CI 0.049–0.069), SRMR = 0.022. All items showed high factor loadings on their intended factor, ranging from 0.75 to 0.92; see Table 1. All factors also showed substantial inter‐factor correlations; see Table 2, in line with the inter‐factor correlations reported in Brotherton et al. (2013). See S1 and S2 Tables for all items and instructions for the original version and Swedish translation of the GCBS.

**TABLE 1:** GCBS scale items and factor loadings.

Scale item	Factor				
	GM	MG	ET	PW	CI
GCBS 1	0.80				
GCBS 6	0.81				
GCBS 11	0.86				
GCBS 2		0.87			
GCBS 7		0.82			
GCBS 12		0.92			
GCBS 3			0.83		
GCBS 8			0.88		
GCBS 13			0.88		
GCBS 4				0.83	
GCBS 9				0.75	
GCBS 14				0.82	
GCBS 5					0.84
GCBS 10					0.76
GCBS 15					0.80

*Note:* *N* = 430.

Abbreviations: CI, control of information; ET, extraterrestrial cover‐up; GM, government malfeasance; MG, malevolent global conspiracies; PW, personal wellbeing.

**TABLE 2:** Factor correlations.

Factor	GM	MG	ET	PW	CI
GM		0.79	0.54	0.76	0.80
MG			0.59	0.83	0.79
ET				0.69	0.56
PW					0.81
CI					

*Note:* *N* = 430. All correlations are significant at the level *p* < 0.001 (2‐tailed).

Abbreviations: CI, control of information; ET, extraterrestrial cover‐up; GM, government malfeasance; MG, malevolent global conspiracies; PW, personal wellbeing.

Reliability estimates, Cronbach's *α*, indicates good internal consistency for all five subscales of the GCBS, GM *α* = 0.87, MG *α* = 0.90, ET *α* = 0.89, PW *α* = 0.84, and CI *α* = 0.84. Cronbach's *α* for the total scale was 0.96.

### 3.2 Latent Profile Analysis

In the next step of the analyses, we conducted a Latent Profile Analysis using MPlus version 8. We selected a model containing four latent profiles, based on model fit statistics, entropy, smallest estimated class proportions (see Table 3), and also conceptual meaning. The LMR test indicated significant improvements in model fit when adding profiles up to four latent profiles, while a 5‐profile model did not improve fit compared to a 4‐profile model. Values for AIC, BIC, and SABIC decreased successively for models with added profiles but with rather small decreases from the 4‐ to 5‐profile model, indicating diminishing returns from adding a fifth class (Nylund‐Gibson and Choi 2018). Also, for the 4‐profile model, the smallest estimated class proportion was 8.6% (*n* = 37), but for the 5‐profile model it was below the stipulated cut‐off with only 3.72% of the participants in the smallest class (*n* = 16). The 4‐profile model also offered distinct, non‐overlapping profiles that differed in degree of conspiracy beliefs, thus providing easily interpretable and meaningful profiles.

**TABLE 3:** Model fit summary for Latent profile analysis.

Model	Log likelihood	AIC	BIC	SABIC	Entropy	Smallest class %	LMR *p*‐value	LMR meaning
1	−3296.450	6612.900	6653.538	6621.804				
2	−2641.924	5315.847	5380.868	5330.093	0.935	36.74	< 0.001	2 > 1
3	−2461.064	4966.128	5055.532	4985.716	0.888	25.58	0.0098	3 > 2
4	−2355.347	4766.694	4880.480	4791.625	0.908	8.60	0.0002	4 > 3
5	−2321.946	4711.891	4850.060	4742.164	0.913	3.72	0.1332	5 < 4

Table 4 presents the mean scores for the four profiles over the five subscales of the GCBS. As can be seen in Figure 1 the class lines for the four profiles do not cross indicating uniform response patterns across the profiles, with the profiles differing in a consistent fashion regarding degree of belief in the measured conspiracy subscales.

**TABLE 4:** Means and standard deviations for the conspiracy beliefs subscales stratified by latent profiles.

Conspiracy subscale	Profile 1	Profile 2	Profile 3	Profile
	*M* (SD)	*M* (SD)	*M* (SD)	*M* (SD)
GM	1.50 (0.87)	2.36 (0.79)	3.39 (0.80)	4.43 (0.88)
MG	1.19 (0.35)	2.36 (1.21)	3.32 (0.76)	4.64 (0.72)
ET	1.20 (0.50)	1.70 (0.86)	2.66 (1.18)	3.10 (1.42)
PW	1.27 (0.44)	2.07 (0.88)	3.33 (0.97)	4.34 (0.82)
CI	1.82 (1.09)	2.97 (0.69)	3.95 (0.90)	4.71 (0.35)

*Note:* *N* = 430.

Abbreviations: CI, control of information; ET, extraterrestrial cover‐up; GM, government malfeasance; MG, malevolent global conspiracies; PW, personal wellbeing.

**FIGURE 1:**
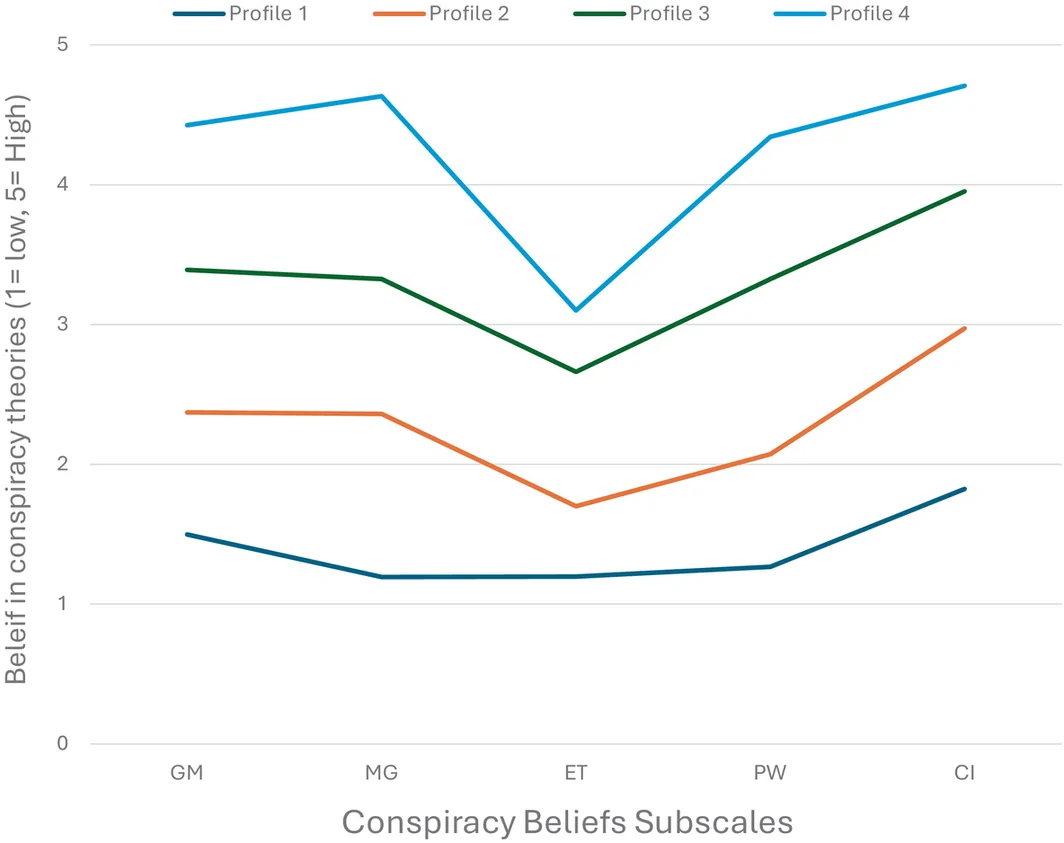
Estimated mean profiles for the subscales in GCBS. *N* = 430. CI, control of information; ET, extraterrestrial cover‐up; GM, government malfeasance; MG, malevolent global conspiracies; PW, personal well‐being.

Profile 1 (*n* = 164, 38.14%) was the largest class and consisted of participants that showed the lowest belief in conspiracy theories across all five subscales of the GCBS. Profile 2 (*n* = 124, 28.84%) was the second largest class, and consisted of participants that showed consistently slightly higher belief in conspiracy theories compared to Profile 1. Profile 3 (*n* = 105, 24.42%) was the third largest class and consisted of participants that evinced the second highest level of belief in conspiracy theories with mean scores of between 3 and 4 on the scale from 1 to 5 (with scores on ET being slightly below the midpoint of 3 on the scale). Profile 4 (*n* = 37, 8.61%) was the smallest class but showed the consistently highest level of conspiracy beliefs over all five subscales compared to the other profiles, with mean scores of between 4 and 5 on the scale from 1 to 5 (with scores on ET being slightly lower at just above the midpoint of 3 on the scale).

### 3.3 Analysis of Auxiliary Variables

Means and standard deviations for the four identified latent profiles across the measured auxiliary variables are presented in Table 5. Effect sizes (Cohen's *d*) presented below are based on these means and standard deviations.

**TABLE 5:** Means (standard deviations) for auxiliary variables stratified by latent profiles.

Auxiliary variables	Profile 1	Profile 2	Profile 3	Profile 4
	*M* (SD)	*M* (SD)	*M* (SD)	*M* (SD)
Experiential ability	3.31 (0.63)	3.40 (0.56)	3.55 (0.61)	3.75 (0.55)
Experiential engagement	3.23 (0.70)	3.24 (0.61)	3.38 (0.78)	3.54 (0.55)
Rational ability	3.75 (0.65)	3.63 (0.67)	3.58 (0.73)	3.99 (0.78)
Rational engagement	3.74 (0.67)	3.42 (0.70)	3.43 (0.70)	3.75 (0.70)
Climate science	4.47 (0.63)	4.04 (1.17)	3.75 (0.93)	2.86 (1.27)
Vaccination science	4.41 (0.72)	3.85 (0.84)	3.27 (0.96)	2.09 (0.95)
CRT correct	2.61 (1.47)	2.43 (1.49)	2.35 (1.57)	2.39 (1.23)
CRT intuitive	1.12 (1.33)	1.31 (1.29)	1.37 (1.36)	1.33 (1.22)
Age	34.75 (16.55)	28.48 (17.22)	27.29 (16.24)	39.47 (20.33)

*Note:* *N* = 430.

Experiential Ability differed across profiles (*χ* ^2^(3) = 24.35, *p* < 0.001). There was no significant difference between Profiles 1 and 2 (*χ* ^2^(3) = 1.83, *p* = 0.176, Cohen's *d* = 0.15). But Profile 1 showed significantly lower self‐reported Experiential Ability than both Profile 4 (*χ* ^2^(3) = 18.25, *p* < 0.001, *d* = 0.71) and Profile 3 (*χ* ^2^(3) = 10.37, *p* = 0.001, *d* = 0.39). Further, Profile 2 showed significantly lower self‐reported Experiential Ability than Profile 4 (*χ* ^2^(3) = 11.04, *p* = 0.001, *d* = 0.63) but did not differ significantly from Profile 3 (*χ* ^2^(3) = 3.51, *p* = 0.061, *d* = 0.26). There was no significant difference between Profile 3 and 4 (*χ* ^2^(3) = 2.95, *p* = 0.086, *d* = 0.33).

Experiential Engagement differed across profiles, *χ* ^2^(3) = 11.29, *p* = 0.010. Profile 4 showed significantly higher self‐reported Experiential Engagement than Profile 1 (*χ* ^2^(3) = 8.43, *p* = 0.004, *d* = 0.46) and Profile 2 (*χ* ^2^(3) = 7.85, *p* = 0.005, *d* = 0.50). There were no significant differences between Profile 3 and any of the other profiles (*p*s > 0.10, *d*s ≤ 0.22). There was no significant difference between Profile 1 and 2 (*p* = 0.887, *d* = 0.02). Thus, the profile evincing the highest belief in all measured types of conspiracy theories also reported significantly higher engagement in intuitive thinking than the two profiles evincing the lowest degree of conspiracy beliefs.

Rational Ability differed across profiles, *χ* ^2^(3) = 9.90, *p* = 0.019. Both Profile 4 (*χ* ^2^(3) = 7.41, *p* = 0.007, *d* = 0.55) and Profile 1 (*χ* ^2^(3) = 3.88, *p* = 0.049, *d* = 0.25) showed significantly higher self‐reported Rational Ability than Profile 3. Further, Profile 4 reported significantly higher Rational Ability than Profile 2 (*χ* ^2^(3) = 6.53, *p* = 0.011, *d* = 0.52). There were no significant differences between Profile 1 and 2 (*p* = 0.139, *d* = 0.18), Profile 1 and 4 (*p* = 0.082, *d* = 0.36), or Profile 2 and 3 (*p* = 0.603, *d* = 0.07). This indicates a non‐linear association between conspiracy beliefs and self‐reported ability to think analytically, with both the profiles reporting the lowest and highest conspiracy beliefs reporting higher analytic ability compared to the other profiles.

Rational Engagement differed across profiles, *χ* ^2^(3) = 21.16, *p* < 0.001. Profiles 1 and 4 reported higher Rational Engagement than the other profiles. Profile 1 had significantly higher self‐reported Rational Engagement than Profile 2 (*χ* ^2^(3) = 13.54, *p* < 0.001, *d* = 0.47), and Profile 3 (*χ* ^2^(3) = 12.67, *p* < 0.001, *d* = 0.46). Likewise, Profile 4 showed significantly higher self‐reported Rational Engagement than Profile 2 (*χ* ^2^(3) = 6.13, *p* = 0.013, *d* = 0.48), and Profile 3 (*χ* ^2^(3) = 5.20, *p* = 0.023, *d* = 0.46). There were no significant differences between Profiles 1 and 4 (*p* = 0.935, *d* = 0.02) or Profiles 2 and 3 (*p* = 0.914, *d* = 0.01). This means that the profiles with both the lowest and highest belief in conspiracy theories reported significantly higher reliance on and enjoyment of analytical and logical thinking than the other two profiles, indicating a non‐linear association between self‐reported enjoyment of analytical and logical thinking and belief in conspiracy theories.

Acceptance of Climate science differed across profiles, *χ* ^2^(3) = 103.27, *p* < 0.001. Here Profile 1 reported significantly higher acceptance of climate science than all the other profiles: Profile 2 (*χ* ^2^(3) = 12.55, *p* < 0.001, *d* = 0.48), Profile 3 (*χ* ^2^(3) = 48.10, *p* < 0.001, *d* = 0.95), and Profile 4 (*χ* ^2^(3) = 56.17, *p* < 0.001, *d* = 2.05). Profile 2 reported higher acceptance of climate science compared to Profile 3 (*χ* ^2^ = 4.02, *p* = 0.044, *d* = 0.27) and Profile 4 (*χ* ^2^(3) = 25.70, *p* < 0.001, *d* = 0.99). Finally, Profile 3 showed significantly higher acceptance of climate science than Profile 4 (*χ* ^2^(3) = 14.54, *p* < 0.001, *d* = 0.87). Overall, acceptance of climate science decreased monotonically as conspiracy belief increased across profiles.

Acceptance of vaccination science also differed across profiles, *χ* ^2^(3) = 274.32, *p* < 0.001. As with climate science, Profile 1 reported significantly higher acceptance of vaccine science than all the other profiles: Profile 2 (*χ* ^2^(3) = 32.37, *p* < 0.001, *d* = 0.72), Profile 3 (*χ* ^2^(3) = 108.52, *p* < 0.001, *d* = 1.39), and Profile 4 (*χ* ^2^(3) = 195.40, *p* < 0.001, *d* = 3.03). Profile 2 reported higher acceptance of vaccination science compared to Profile 3 (*χ* ^2^(3) = 22.22, *p* < 0.001, *d* = 0.65) and Profile 4 (*χ* ^2^(3) = 104.17, *p* < 0.001, *d* = 2.03). Lastly, Profile 3 reported significantly higher acceptance of vaccination science compared to Profile 4 (*χ* ^2^(3) = 39.24, *p* < 0.001, *d* = 1.23). Once again, when comparing the four profiles, the results show a significantly lower degree of acceptance of vaccination science as conspiracy beliefs increase.

Regarding the performance‐based measures of analytical thinking, neither CRT Correct (*χ* ^2^(3) = 2.27, *p* = 0.518, *d*s ≤ 0.17) nor CRT Intuitive (*χ* ^2^(3) = 2.73, *p* = 0.436, *d*s ≤ 0.19) differed significantly across profiles.

Finally, Age differed significantly across profiles, *χ* ^2^(3) = 21.81, *p* < 0.001. Profile 1 was significantly older than Profiles 2 (*χ* ^2^(3) = 8.99, *p* = 0.003, *d* = 0.37) and 3 (*χ* ^2^(3) = 13.37, *p* < 0.001, *d* = 0.45). Profile 4 was also significantly older than Profiles 2 (*χ* ^2^(3) = 8.92, *p* = 0.003, *d* = 0.61) and 3 (*χ* ^2^(3) = 10.18, *p* = 0.001, *d* = 0.70). There were no significant differences between Profiles 1 and 4 (*p* = 0.189, *d* = 0.27) or Profiles 2 and 3 (*p* = 0.603, *d* = 0.07). This means that the profiles with both the lowest and highest belief in conspiracy theories were significantly older than the other two profiles, indicating a non‐linear association between age and belief in conspiracy theories.

## 4 Discussion

The aim of the present study was to (i) use latent profile analysis to identify latent profiles, or groups of participants, that share meaningful response patterns regarding beliefs in conspiracy theories, and (ii) analyze differences in auxiliary variables, using the BHC method, between the identified latent profiles including the following variables: Experiential Ability, Experiential Engagement, Rational Ability, Rational Engagement, Acceptance of Climate science, Acceptance of Vaccination science, CRT correct and CRT Intuitive.

Importantly, conspiracy beliefs and the rejection of scientific consensus may be understood as a multifaceted phenomenon shaped by a complex interplay of cognitive, motivational, ideological, and socio‐political factors (e.g., Adam‐Troian et al. 2023; Biddlestone et al. 2022; Bowes et al. 2023). The present study was not designed to provide a comprehensive account of these broader dynamics, but rather to examine whether distinct latent profiles could be identified based on generic conspiracy beliefs and individual differences in thinking styles. Thus, our interpretations should be understood within the cognitive scope of the current investigation. Latent profile analysis resulted in a model with four distinct profiles that showed consistent, and distinct, response patterns over all five GCBS subscales, with no overlap between the profiles. Thus, consistency was manifested in the response patterns of all four latent profiles, regardless of whether the conspiracy theories concerned belief in routine governmental malfeasance, belief in the existence of malevolent global conspiracies, belief in the existence and cover‐up of extraterrestrials, concerns over the unethical control of information, or belief in conspiracies infringing on personal well‐being and liberty. The profiles differed, in a consistent fashion, in the degree of belief in conspiracy theories. Profile 1, the largest profile identified, evinced the lowest degree of belief in all five types of conspiracy theories, with all means between 1 (“definitely not true”) and 2 (“probably not true”) on a 5‐point scale. The second largest profile, Profile 2, reported slightly higher endorsement of all types of conspiracy theories, compared with Profile 1, but with all means below the mid‐point (between “probably not true” and “not sure/cannot decide”). The third largest profile, Profile 3, indicated higher endorsement of all conspiracy theories, with mean endorsements between 3 (“not sure/cannot decide”) and 4 (“probably true”). Finally, the smallest profile, Profile 4, reported the highest degree of belief in all five types of conspiracy theories, with means between 4 (“probably true”) and 5 (“definitely true”) on the 5‐point scale (with belief in the existence and cover‐up of extraterrestrials slightly lower at the midpoint of the scale).

In line with Frenken and Imhoff (2021), the results of this study showed that belief in extraterrestrial cover‐up conspiracy theories are in general lower compared to the other types of conspiracy theories for all profiles except Profile 1 (possibly due to floor effects as profile 1 report overall very low beliefs). This may be due to a conceptual difference between extraterrestrial cover‐up conspiracy theories and the other types measured by the GCBS, in terms of there being a more explicit element of harm or oppression of the public in the other types of conspiracy theories compared to extraterrestrial cover‐up (Castanho Silva et al. 2017). Further, and related to the above, the items measuring belief in extraterrestrial cover‐up conspiracy theories are also framed in a way that makes it less obvious, compared to items measuring the other types of conspiracy theories, why there is a need for a conspiracy in the first place (i.e., what is it that potential conspirators gain by keeping evidence hidden from the public concerning for instance contact with aliens).

More importantly, these results are also in line with the results reported by Agley and Xiao (2021), Frenken and Imhoff (2021), and Jones et al. (2023), who also found distinct, and (largely) non‐overlapping, profiles with consistent response patterns regarding beliefs in conspiracy theories. Further, as reported in the previous studies using LPA, we also find that the profile reporting the highest degree of conspiracy beliefs is the smallest profile with 8.61% of the participants, which is rather close to the numbers presented by Agley and Xiao (2021, 8.18%), Frenken and Imhoff (2021, varying between 5.5% and 7.5% in the datasets analyzed), and Jones et al. (2023, 15.3%). As discussed by Frenken and Imhoff (2021), this manifested consistency of response patterns in relation to different types of conspiracy theories further strengthens the notion of a conspiracy mindset. But, as Frenken and Imhoff also emphasize, the mindset notion does not constitute an “explanatory construct” (2021, 11), and more research is needed to explicate which factors are related to differences in these consistent response patterns concerning belief in conspiracy theories. We therefore analyzed differences between the identified profiles in our study, in several auxiliary variables, using a person‐centered approach rather than the hitherto more common variable‐centered approach.

Several previous studies (e.g., Alper et al. 2021; Jedinger et al. 2023; Ozono and Sakakibara 2024; Stanley et al. 2021) have indicated that performance on the CRT is related to belief in conspiracy theories. However, in the present study there were no differences between any of the identified profiles in the two CRT variables analyzed, indicating that differences between profiles endorsing conspiracy theories to various degrees is not related to this performance‐based measure of analytical thinking. Even if these results are not in line with previous studies reporting negative correlations between performance on the CRT and conspiracy beliefs (e.g., Alper et al. 2021; Jedinger et al. 2023; Ozono and Sakakibara 2024; Stanley et al. 2021), other studies suggest a more complex association between conspiracy belief and CRT performance. For instance, Hattersley et al. (2022) found significant negative correlations between performance on the CRT and belief in conspiracy theories deemed implausible by participants in a pilot‐study but non‐significant correlations for plausible conspiracy theories (operationalized as being evaluated as above the midpoint on a 10‐point plausibility scale). To the extent that the types of conspiracy theories included in the GCBS can be considered plausible this may explain our results. Further, Ståhl and Van Prooijen (2018) found that performance on the CRT was related to conspiracy beliefs only for participants that also valued epistemic rationality, which we did not control for in our study. Finally, as pointed out in previous studies (e.g., Sirota et al. 2021), there is a debate concerning to what extent some versions of the CRT test measure mathematical ability rather than analytical thinking ability. To a large extent, CRT items, both the original items created by Frederick (2005) and other versions, are mathematical. Therefore, these types of CRT items also capture mathematical ability besides the propensity to engage in analytical thinking (Pennycook and Ross 2016; Sirota et al. 2021). However, evidence shows that the CRT is predictive of beliefs without theoretical relation to numeracy (e.g., religious beliefs), and also predictive of outcomes after controlling for mathematical ability (Pennycook and Ross 2016), which indicates that the CRT is not just a measure of mathematical ability but also captures the propensity to engage in analytical thinking in order to override intuitive, incorrect, answers. For this study we pilot tested several CRT items and used four items that seemed best suited regarding the risk for floor or ceiling effects as well as ease of understanding. However, these items all contain a mathematical aspect so there is a risk for confounding mathematical ability with propensity for analytical thinking to some extent.

We also included subjective, self‐report, measures of analytical and intuitive thinking using the four subscales from the Rational‐Experiential inventory (REI). This to assess participants' self‐perceived reliance on and ability in intuitive and analytical thinking, rather than their actual more objective performance on the CRT test. Interestingly, we found a non‐linear association between both subscales measuring analytical thinking and conspiracy beliefs, with the profiles reporting both the highest and lowest degree of conspiracy beliefs reporting higher reliance on and ability in analytical thinking compared to the profiles reporting more moderate beliefs. In contrast, when it comes to perceived reliance on and ability in intuitive thinking our results indicate more of a linear association with profiles reporting higher degrees of conspiracy beliefs also reporting higher degrees of reliance on and ability in intuitive thinking compared to profiles reporting lower conspiracy beliefs.

What separates the profiles reporting the highest and lowest belief in conspiracy theories, regarding thinking styles, is that the profiles with higher degrees of conspiracy beliefs report higher reliance on, and ability in, intuitive thinking. This is in line with previous studies reporting positive associations between self‐reported intuitive thinking and belief in conspiracy theories (e.g., Georgiou et al. 2019; Pytlik et al. 2020; Swami et al. 2014). However, the finding that the two profiles showing the lowest and highest degree of belief in conspiracy theories respectively (Profile 1 and 4), also showed higher degrees of self‐reported ability and engagement in analytical thinking compared with the two other profiles is not in line with the results from several previous studies that indicate a negative association between self‐reported analytical thinking and belief in conspiracy theories (e.g., Georgiou et al. 2019; Gligorić et al. 2021; Jastrzębski and Chuderski 2022; Ozono and Sakakibara 2024; Swami and Barron 2021). However, our results are in line with the results presented by Jones et al. (2023), that reported higher degree of self‐reported analytical thinking in the profiles evincing the lowest and highest belief in conspiracy theories concerning Covid‐19 (and lower analytical thinking in the profile with moderate conspiracy belief).

These results, from our study as well as Jones et al. (2023), are interesting as it has been argued that a higher degree of reliance on analytical, and less reliance on intuitive, thinking is related to lower belief in conspiracy thinking. This is because of the fact that analytical, type 2, thinking is associated with less reliance on heuristics and cognitive biases, less reliance on “gut feelings” and affective reasoning, a greater ability to identify logical fallacies and errors in reasoning, and, therefore, better evaluation of support and evidence for a conclusion, which should render (largely) unsupported conspiracy theories less believable (e.g., Binnendyk and Pennycook 2022; Pennycook et al. 2015a; Ståhl and Van Prooijen 2018; Swami et al. 2014; van Prooijen 2017; Yelbuz et al. 2022).

Therefore, it is surprising that the profile evincing the highest endorsement of conspiracy theories also reported high perceived ability, and engagement, in both analytical and intuitive thinking. However, Van Prooijen et al. (2020) argue that both intuitive Type 1 thinking, and analytical Type 2 thinking, contribute to belief in conspiracy theories. According to Van Prooijen et al., initial belief in conspiracy theories originate through intuitive Type 1 thinking, whereas analytical Type 2 thinking can be used to justify and maintain these beliefs (via, for instance, motivated reasoning and selective search for evidence) (see also Brashier 2023). Possibly then, according to Van Prooijens et al. reasoning, a higher self‐reported reliance on, and trust in one's ability for, intuitive thinking may impart a higher susceptibility to forming conspiracy beliefs in Profiles 3 and 4, while perceived reliance on, and ability in, analytical thinking, especially in Profile 4, could be used to maintain conspiracy beliefs once developed. This type of reasoning is also in line with the Three‐stage dual process model, suggested by Pennycook and colleagues (e.g., Pennycook 2023; Pennycook et al. 2015b), according to which analytical thinking can be used for deliberation in order to reduce errors (and override incorrect responses, heuristics etc.). But, importantly, analytical thinking may also, according to the three‐stage dual process model, be used to rationalize, that is justify and maintain, current beliefs rather than question its veracity. This line of reasoning constitutes an interesting, possible, albeit speculative, explanation for the results presented in this study and Jones et al. (2023) that merits further research, preferably using more objective measures of participants' actual use of intuitive and analytical thinking rather than self‐report measures. It should also be acknowledged that some of the outcomes measured in this study, that is acceptance of climate and vaccination science, also are ideologically grounded which could also affect how participants report their thinking styles. However, these types of variables were not included in this study, nor in line with our aim, so we cannot assess this explanation further.

Finally, both acceptance of climate and vaccination science differed between the four identified profiles. Here the results indicated significant differences between all four profiles, where profiles evincing higher degrees of beliefs in conspiracy theories reported significantly less acceptance of both climate and vaccination compared with profiles showing less belief in conspiracy theories. These findings are in line with previous studies (e.g., Lewandowsky, Gignac, et al. 2013; Lewandowsky, Oberauer, et al. 2013; Marques et al. 2021). Agley and Xiao (2021) also reported that trust in science was a strong predictor of profile membership in their study, where lower trust in science was related to higher belief in conspiracy theories. Several mechanisms have been suggested as possible explanations of the relationship between trust in science and belief in conspiracy theories, such as trust and distrust in authorities, reliance on heuristics in thinking, reasoning errors and cognitive biases, less analytical thinking, and reliance on intuitive thinking (e.g., Biddlestone et al. 2022; Lewandowsky and Oberauer 2016; Marques et al. 2021; Rutjens and Većkalov 2022). As argued by Van Prooijen et al. (2020), it is possible that both intuitive Type 1 thinking and analytical Type 2 thinking plays a role in science distrust in the same manner as suggested concerning conspiracy beliefs. That is, distrust in science may originate through Type 1 thinking, but analytical Type 2 thinking may be used to justify and maintain these beliefs, through for instance motivated reasoning and selective search for evidence. This, again, posits a possible explanation for the finding that Profile 4, with both the highest degree of conspiracy belief and the lowest degree of science acceptance, also reports high ability and engagement in analytical thinking. Although not a specific aim of this study, we also included age as an auxiliary variable. As we included both a younger high school sample and an older general sample in our study, we wanted to investigate to what extent the samples included constituted separate profiles identified in the LPA. Age differed significantly between the profiles, with the profiles reporting the highest and lowest belief in conspiracy theories being older than the two profiles showing more moderate beliefs. These results indicate a non‐linear association between age and belief in conspiracy theories, which is not fully in line with results presented in the meta‐analysis by Bordeleau and Stockemer (2024), which reported finding a robust, albeit weak, negative association with younger age being related to higher conspiracy beliefs (see also Douglas et al. 2019; Enders et al. 2024). Looking only at the first three profiles identified in our study the trend is that profiles showing higher conspiracy beliefs tend to be younger than profiles showing lower conspiracy beliefs, consistent with a linear negative association between age and conspiracy beliefs. However, Profile 4, that reports the highest degree of conspiracy beliefs, is also the profile with the highest mean age (although not significantly older than Profile 1). This type of finding testifies to the value of utilizing non‐linear, and person‐centered, approaches to enable a more nuanced understanding of this phenomenon.

### 4.1 Methodological Issues

There are several limitations that need to be addressed in the present study. One limitation of the present study is that profile membership was not examined in relation to a broader set of external predictors beyond age. Variables such as education, socioeconomic status, political ideology, and institutional trust may help clarify what differentiates these profiles beyond the cognitive indicators. Also, we used self‐report for all measured variables which increases the risk for common method biases (CMBs) (e.g., Podsakoff et al. 2012, 2024). In an effort to reduce such biases, we followed the recommendations of Podsakoff et al. (2012), by clearly explaining the purpose of the study and how the information they provided would be used, we also tried to make the written information about the study, as well as the instructions for the questionnaires, as distinct and clear as possible. However, as described in the method section we specifically avoided using terms such as “conspiracy theory”, as these kinds of terms may have a pejorative meaning to participants, in the information given in order not to increase the risk of response biases such as demand characteristics and social desirability. Another limitation is that all measurements, except for the CRT, used Likert‐type scales which also increases the risk for common method bias. However, and in line with recommendations by Podsakoff et al. (2012), the anchor labels for the scales where not identical for all measurements, and we also used labels for all points on the scales (and not just the endpoints) to decrease ambiguity in interpretation of the different points on the scales. A third limitation is the use of an online survey for about half of the participants, which means that any ambiguities or problems experienced by these participants could not be addressed. For the high school sample, one of us (Peter Karlsson) was present for all measurement occasions and could therefore answer questions and clarify any ambiguous items. As explained by Podsakoff et al. (2012), ambiguous, and difficult, items increase the risk of participants relying on their own systematic response patterns (e.g., acquiescing, or midpoint response styles). In order to minimize the risk of participants experiencing items as ambiguous or overly difficult we conducted a pilot study in order to identify instruments, and items for the CRT measurement, that were too difficult or ambiguous. Finally, in this study we used convenience sampling methods, which means that our sample is not representative of the general population.

## 5 Conclusion

Using Latent Profile Analysis (LPA) we identified four distinct profiles that showed consistent and distinct response patterns over all five subscales of the Generic Conspiracy Belief Scale (GCBS). There was no overlap between any of the profiles, indicating uniform response patterns across the profiles, with the profiles differing in a consistent fashion regarding degree of belief in the measured conspiracy subscales, ranging from low (Profile 1) to strong conspiracy beliefs (Profile 4). Further analyses of differences in auxiliary variables, using the BHC method, indicated several significant differences between the latent profiles. Overall, less belief in conspiracy theories was associated with less self‐reported ability and engagement in intuitive thinking and higher acceptance of climate and vaccine science. Somewhat unexpectedly, participants who held the strongest conspiracy beliefs (Profile 4) as well as those who held the lowest beliefs in conspiracy theories (Profile 1) reported higher levels of analytical thinking, compared with the other two profiles, suggesting that people who most strongly endorse conspiracy theories report high use of both analytical and intuitive thinking styles. A potential explanation for this result may be that conspiracy beliefs originate through intuitive thinking while analytical thinking can be used to maintain and justify these beliefs (through, for instance, motivated reasoning). Overall, the present findings contribute to a cognitive understanding of conspiracy beliefs and science acceptance. However, they should not be interpreted as implying that conspiracy‐related profiles are purely cognitive in nature. Rather, thinking styles likely interact with motivational and socio‐political factors in shaping both conspiracy endorsement as well as rejection of science. Future research combining cognitive profiles with broader contextual predictors will be important for understanding how these processes together contribute to conspiracy beliefs and distrust of science.

Future research should also utilize person‐centered analysis more than has been the case up until now in order to gain a more nuanced understanding of conspiracy beliefs than possible by relying only on more traditional variable‐centered approaches. Further, future research should also directly address the suggestion by Van Prooijen et al. (2020) that belief in conspiracy theories originates from intuitive Type 1 thinking while analytical Type 2 thinking can be used to justify and maintain these beliefs when formed, for instance through selective search for evidence and motivated reasoning. Preferably, this should also be studied experimentally, where the effects of manipulating participants' motivation to use analytical thinking to maintain beliefs versus reaching a more veridical understanding of events on conspiracy beliefs could be evaluated. Finally, in this study we focused on more cognitive factors in relation to conspiracy beliefs. Even though these are important factors, there are several other types of factors that should be considered in future research, such as education, socioeconomic status, and political ideology.

## Author Contributions

Conceptualization: Peter Karlsson, Torbjörn Josefsson, Sara Svensson, and Linus Andersson. Methodology: Peter Karlsson, Torbjörn Josefsson, Sara Svensson, and Linus Andersson. Statistical analysis: Peter Karlsson Writing original draft: Peter Karlsson and Torbjörn Josefsson. Writing – review and editing: Peter Karlsson, Torbjörn Josefsson, Sara Svensson, and Linus Andersson.

## Funding

The authors received funding for the survey among the high school population from the Swedish Research Council (Grant 2021‐06266).

## Ethics Statement

The present study received ethical approval from the Swedish Ethical Review Authority (Dnr 2022‐02402‐01).

## Conflicts of Interest

The authors declare no conflicts of interest.

## Supporting information


**Table S1:** GCBS instructions and items original version.

**Table S2:** GCBS instructions and items Swedish translation.

**Table S3:** CRT items original version and Swedish translation.

**Table S4:** Acceptance of climate and vaccination science items original version.

**Table S5:** Acceptance of climate and vaccination science items Swedish translation.

## Data Availability

The datafile is available at osf.io: https://doi.org/10.17605/OSF.IO/X5TP4.

## References

[sjop70120-bib-0001] Adam‐Troian, J. , M. Chayinska , M. P. Paladino , Ö. M. Uluğ , J. Vaes , and P. Wagner‐Egger . 2023. “Of Precarity and Conspiracy: Introducing a Socio‐Functional Model of Conspiracy Beliefs.” British Journal of Social Psychology 62: 136–159. 10.1111/bjso.12597. PMC10100481 36366839

[sjop70120-bib-0002] Agley, J. , and Y. Xiao . 2021. “Misinformation About COVID‐19: Evidence for Differential Latent Profiles and a Strong Association With Trust in Science.” BMC Public Health 21, no. 1: 89. 10.1186/s12889-020-10103-x. PMC7789893 33413219

[sjop70120-bib-0003] Alper, S. , F. Bayrak , and O. Yilmaz . 2021. “Psychological Correlates of COVID‐19 Conspiracy Beliefs and Preventive Measures: Evidence From Turkey.” Current Psychology 40, no. 11: 5708–5717. 10.1007/S12144-020-00903-0. PMC7324005 32837129

[sjop70120-bib-0004] Asparouhov, T. , and B. Muthén . 2014. “Auxiliary Variables in Mixture Modeling: Using the BCH Method in Mplus to Estimate a Distal Outcome Model and an Arbitrary Secondary Model.” Mplus Web Notes 21, no. 2: 1–22. https://www.statmodel.com/examples/webnotes/webnote21.pdf.

[sjop70120-bib-0005] Bartlett, J. , and C. Miller . 2010. The Power of Unreason: Conspiracy Theories, Extremism and Counter‐Terrorism. Demos.

[sjop70120-bib-0006] Biddlestone, M. , F. Azevedo , and S. Van Der Linden . 2022. “Climate of Conspiracy: A Meta‐Analysis of the Consequences of Belief in Conspiracy Theories About Climate Change.” Current Opinion in Psychology 46: 101390. 10.1016/j.copsyc.2022.101390. 35802986

[sjop70120-bib-0007] Binnendyk, J. , and G. Pennycook . 2022. “Intuition, Reason, and Conspiracy Beliefs.” Current Opinion in Psychology 47: 101387. 10.1016/j.copsyc.2022.101387. 35785571

[sjop70120-bib-0008] Björklund, F. , and M. Bäckström . 2008. “Individual Differences in Processing Styles: Validity of the Rational–Experiential Inventory.” Scandinavian Journal of Psychology 49, no. 5: 439–446. 10.1111/j.1467-9450.2008.00652.x. 18452500

[sjop70120-bib-0009] Bordeleau, J. N. , and D. Stockemer . 2024. “On the Relationship Between Age and Conspiracy Beliefs.” Political Psychology 46: 931–946. 10.1111/pops.13044.

[sjop70120-bib-0010] Bowes, S. M. , T. H. Costello , and A. Tasimi . 2023. “The Conspiratorial Mind: A Meta‐Analytic Review of Motivational and Personological Correlates.” Psychological Bulletin 149, no. 5–6: 259–293. 10.1037/bul0000392. 37358543

[sjop70120-bib-0011] Brashier, N. M. 2023. “Do Conspiracy Theorists Think Too Much or Too Little?” Current Opinion in Psychology 49: 101504. 10.1016/j.copsyc.2022.101504. 36577227

[sjop70120-bib-0012] Brotherton, R. , and C. C. French . 2014. “Belief in Conspiracy Theories and Susceptibility to the Conjunction Fallacy.” Applied Cognitive Psychology 28, no. 2: 238–248. 10.1002/acp.2995.

[sjop70120-bib-0013] Brotherton, R. , C. C. French , and A. D. Pickering . 2013. “Measuring Belief in Conspiracy Theories: The Generic Conspiracist Beliefs Scale.” Frontiers in Psychology 4: 279. 10.3389/fpsyg.2013.00279. PMC3659314 23734136

[sjop70120-bib-0014] Brown, T. A. 2015. Confirmatory Factor Analysis for Applied Research. Guilford publications.

[sjop70120-bib-0015] Cassese, E. C. , C. E. Farhart , and J. M. Miller . 2020. “Gender Differences in COVID‐19 Conspiracy Theory Beliefs.” Politics & Gender 16, no. 4: 1009–1018. 10.1017/S1743923X20000409.

[sjop70120-bib-0016] Castanho Silva, B. , F. Vegetti , and L. Littvay . 2017. “The Elite Is up to Something: Exploring the Relation Between Populism and Belief in Conspiracy Theories.” Swiss Political Science Review 23, no. 4: 423–443. 10.1111/spsr.12270.

[sjop70120-bib-0017] Diethelm, P. , and M. McKee . 2009. “Denialism: What Is It and How Should Scientists Respond?” European Journal of Public Health 19, no. 1: 2–4. 10.1093/eurpub/ckn139. 19158101

[sjop70120-bib-0018] Douglas, K. M. , and R. M. Sutton . 2015. “Climate Change: Why the Conspiracy Theories Are Dangerous.” Bulletin of the Atomic Scientists 71, no. 2: 98–106. 10.1177/0096340215571908.

[sjop70120-bib-0019] Douglas, K. M. , and R. M. Sutton . 2018. “Why Conspiracy Theories Matter: A Social Psychological Analysis.” European Review of Social Psychology 29, no. 1: 256–298. 10.1080/10463283.2018.1537428.

[sjop70120-bib-0020] Douglas, K. M. , and R. M. Sutton . 2023. “What Are Conspiracy Theories? A Definitional Approach to Their Correlates, Consequences, and Communication.” Annual Review of Psychology 74, no. 1: 271–298. 10.1146/annurev-psych-032420-031329. 36170672

[sjop70120-bib-0021] Douglas, K. M. , J. E. Uscinski , R. M. Sutton , et al. 2019. “Understanding Conspiracy Theories.” Political Psychology 40: 3–35. 10.1111/pops.12568.

[sjop70120-bib-0022] Drinkwater, K. G. , N. Dagnall , A. Denovan , and N. Neave . 2020. “Psychometric Assessment of the Generic Conspiracist Beliefs Scale.” PLoS One 15, no. 3: e0230365. 10.1371/journal.pone.0230365. PMC7082021 32191741

[sjop70120-bib-0023] Enders, A. , C. Klofstad , A. Diekman , et al. 2024. “The Sociodemographic Correlates of Conspiracism.” Scientific Reports 14, no. 1: 14184. 10.1038/s41598-024-64098-1. PMC11190281 38902264

[sjop70120-bib-0024] Evans, J. S. B. 2008. “Dual‐Processing Accounts of Reasoning, Judgment, and Social Cognition.” Annual Review of Psychology 59, no. 1: 255–278. 10.1146/annurev.psych.59.103006.093629. 18154502

[sjop70120-bib-0025] Evans, J. S. B. , and K. E. Stanovich . 2013. “Dual‐Process Theories of Higher Cognition: Advancing the Debate.” Perspectives on Psychological Science 8, no. 3: 223–241. 10.1177/1745691612460685. 26172965

[sjop70120-bib-0026] Ferguson, S. L. G. , E. W. Moore , and D. M. Hull . 2020. “Finding Latent Groups in Observed Data: A Primer on Latent Profile Analysis in Mplus for Applied Researchers.” International Journal of Behavioral Development 44, no. 5: 458–468. 10.1177/0165025419881721.

[sjop70120-bib-0027] Frederick, S. 2005. “Cognitive Reflection and Decision Making.” Journal of Economic Perspectives 19, no. 4: 25–42. 10.1257/089533005775196732.

[sjop70120-bib-0028] Frenken, M. , and R. Imhoff . 2021. “A Uniform Conspiracy Mindset or Differentiated Reactions to Specific Conspiracy Beliefs? Evidence From Latent Profile Analyses.” International Review of Social Psychology 34, no. 1: 27. 10.5334/irsp.590.

[sjop70120-bib-0029] Georgiou, N. , P. Delfabbro , and R. Balzan . 2019. “Conspiracy Beliefs in the General Population: The Importance of Psychopathology, Cognitive Style and Educational Attainment.” Personality and Individual Differences 151: 109521. 10.1016/j.paid.2019.109521.

[sjop70120-bib-0030] Georgiou, N. , P. Delfabbro , and R. Balzan . 2021. “Conspiracy Theory Beliefs, Scientific Reasoning and the Analytical Thinking Paradox.” Applied Cognitive Psychology 35, no. 6: 1523–1534. 10.1002/acp.3885.

[sjop70120-bib-0031] Gligorić, V. , M. M. da Silva , S. Eker , et al. 2021. “The Usual Suspects: How Psychological Motives and Thinking Styles Predict the Endorsement of Well‐Known and COVID‐19 Conspiracy Beliefs.” Applied Cognitive Psychology 35, no. 5: 1171–1181. 10.1002/acp.3844. PMC8212084 34177101

[sjop70120-bib-0032] Hansson, S. O. 2013. “Defining Pseudoscience and Science.” In Philosophy of Pseudoscience: Reconsidering the Demarcation Problem, edited by M. Pigliucci and M. Boudry , 61–77. University of Chicago Press.

[sjop70120-bib-0033] Hansson, S. O. 2017. “Science Denial as a Form of Pseudoscience.” Studies in History and Philosophy of Science Part A 63: 39–47. 10.1016/j.shpsa.2017.05.002. 28629651

[sjop70120-bib-0034] Hattersley, M. , G. D. Brown , J. Michael , and E. A. Ludvig . 2022. “Of Tinfoil Hats and Thinking Caps: Reasoning Is More Strongly Related to Implausible Than Plausible Conspiracy Beliefs.” Cognition 218: 104956. 10.1016/j.cognition.2021.104956. 34813995

[sjop70120-bib-0035] Imhoff, R. , T. Bertlich , and M. Frenken . 2022. “Tearing Apart the ‘Evil’ Twins: A General Conspiracy Mentality Is Not the Same as Specific Conspiracy Beliefs.” Current Opinion in Psychology 46: 101349. 10.1016/j.copsyc.2022.101349. 35537265

[sjop70120-bib-0036] Jastrzębski, J. , and A. Chuderski . 2022. “Analytic Thinking Outruns Fluid Reasoning in Explaining Rejection of Pseudoscience, Paranormal, and Conspiracist Beliefs.” Intelligence 95: 101705. 10.1016/j.intell.2022.101705.

[sjop70120-bib-0037] Jedinger, A. , L. Masch , and A. M. Burger . 2023. “Cognitive Reflection and Endorsement of the ‘Great Replacement; Conspiracy Theory.” Social Psychological Bulletin 18: 1–12. 10.32872/spb.10825.

[sjop70120-bib-0038] Jolley, D. , R. Meleady , and K. M. Douglas . 2020. “Exposure to Intergroup Conspiracy Theories Promotes Prejudice Which Spreads Across Groups.” British Journal of Psychology 111, no. 1: 17–35. 10.1111/bjop.12385. PMC7004178 30868563

[sjop70120-bib-0039] Jones, C. , N. Galbraith , D. Boyda , D. B. Martin , and K. Jackson . 2023. “A Latent Profile Analysis of COVID‐19 Conspiracy Beliefs: Associations With Thinking Styles, Mistrust, Socio‐Political Control, Need for Closure and Verbal Intelligence.” Personality and Individual Differences 207: 112155. 10.1016/j.paid.2023.112155. PMC9988712 36923243

[sjop70120-bib-0040] Jylhä, K. M. , S. K. Stanley , M. Ojala , and E. J. Clarke . 2022. “Science Denial: A Narrative Review and Recommendations for Future Research and Practice.” European Psychologist 28, no. 3: 151–161. 10.1027/1016-9040/a000487.

[sjop70120-bib-0041] Lewandowsky, S. , J. Cook , K. Oberauer , S. Brophy , E. A. Lloyd , and M. Marriott . 2015. “Recurrent Fury: Conspiratorial Discourse in the Blogosphere Triggered by Research on the Role of Conspiracist Ideation in Climate Denial.” Journal of Social and Political Psychology 3, no. 1: 142–178. 10.5964/jspp.v3i1.443.

[sjop70120-bib-0042] Lewandowsky, S. , G. E. Gignac , and K. Oberauer . 2013. “The Role of Conspiracist Ideation and Worldviews in Predicting Rejection of Science.” PLoS One 8, no. 10: e75637. 10.1371/journal.pone.0075637. PMC3788812 24098391

[sjop70120-bib-0043] Lewandowsky, S. , and K. Oberauer . 2016. “Motivated Rejection of Science.” Current Directions in Psychological Science 25, no. 4: 217–222. 10.1177/0963721416654436.

[sjop70120-bib-0044] Lewandowsky, S. , K. Oberauer , and G. E. Gignac . 2013. “NASA Faked the Moon Landing‐ Therefore, (Climate) Science Is a Hoax: An Anatomy of the Motivated Rejection of Science.” Psychological Science 24, no. 5: 622–633. 10.1177/0956797612457686. 23531484

[sjop70120-bib-0045] Marques, M. D. , J. R. Kerr , M. N. Williams , M. Ling , and J. McLennan . 2021. “Associations Between Conspiracism and the Rejection of Scientific Innovations.” Public Understanding of Science 30, no. 7: 854–867. 10.1177/09636625211007013. 33860713

[sjop70120-bib-0046] Muthén, L. , and B. Muthén . 2017. Mplus User's Guide [Computer Software Manual]. 8th ed. Muthén & Muthén.

[sjop70120-bib-0047] Nye, C. D. 2023. “Reviewer Resources: Confirmatory Factor Analysis.” Organizational Research Methods 26, no. 4: 608–628. 10.1177/10944281221120541.

[sjop70120-bib-0048] Nylund‐Gibson, K. , and A. Y. Choi . 2018. “Ten Frequently Asked Questions About Latent Class Analysis.” Translational Issues in Psychological Science 4, no. 4: 440–461. 10.1037/tps0000176.

[sjop70120-bib-0049] Oldrati, V. , J. Patricelli , B. Colombo , and A. Antonietti . 2016. “The Role of Dorsolateral Prefrontal Cortex in Inhibition Mechanism: A Study on Cognitive Reflection Test and Similar Tasks Through Neuromodulation.” Neuropsychologia 91: 499–508. 10.1016/j.neuropsychologia.2016.09.010. 27647553

[sjop70120-bib-0050] Ozono, H. , and R. Sakakibara . 2024. “The Moderating Role of Reflective Thinking on Personal Factors Affecting Belief in Conspiracy Theories.” Applied Cognitive Psychology 38, no. 1: e4142. 10.1002/acp.4142.

[sjop70120-bib-0051] Pacini, R. , and S. Epstein . 1999. “The Relation of Rational and Experiential Information Processing Styles to Personality, Basic Beliefs, and the Ratio‐Bias Phenomenon.” Journal of Personality and Social Psychology 76, no. 6: 972–987. 10.1037/0022-3514.76.6.972. 10402681

[sjop70120-bib-0052] Pennycook, G. 2023. “A Framework for Understanding Reasoning Errors: From Fake News to Climate Change and Beyond.” Advances in Experimental Social Psychology 67: 131–208. 10.1016/bs.aesp.2022.11.003.

[sjop70120-bib-0053] Pennycook, G. , J. A. Cheyne , P. Seli , D. J. Koehler , and J. A. Fugelsang . 2012. “Analytic Cognitive Style Predicts Religious and Paranormal Belief.” Cognition 123, no. 3: 335–346. 10.1016/j.cognition.2012.03.003. 22481051

[sjop70120-bib-0054] Pennycook, G. , J. A. Fugelsang , and D. J. Koehler . 2015a. “Everyday Consequences of Analytic Thinking.” Current Directions in Psychological Science 24, no. 6: 425–432. 10.1177/0963721415604610.

[sjop70120-bib-0055] Pennycook, G. , J. A. Fugelsang , and D. J. Koehler . 2015b. “What Makes Us Think? A Three‐ Stage Dual‐Process Model of Analytic Engagement.” Cognitive Psychology 80: 34–72. https://www.sciencedirect.com/science/article/pii/S0010028515000481?via%3Dihub. 10.1016/j.cogpsych.2015.05.001 26091582

[sjop70120-bib-0081] Pennycook, G. , and R. M. Ross . 2016. “Commentary: Cognitive Reflection vs. Calculation in Decision Making.” Frontiers in Psychology 7: 9. 10.3389/fpsyg.2016.00009. PMC4722428 26834682

[sjop70120-bib-0056] Podsakoff, P. M. , S. B. MacKenzie , and N. P. Podsakoff . 2012. “Sources of Method Bias in Social Science Research and Recommendations on How to Control It.” Annual Review of Psychology 63, no. 1: 539–569. 10.1146/annurev-psych-120710-100452. 21838546

[sjop70120-bib-0057] Podsakoff, P. M. , N. P. Podsakoff , L. J. Williams , C. Huang , and J. Yang . 2024. “Common Method Bias: It's Bad, It's Complex, It's Widespread, and It's Not Easy to Fix.” Annual Review of Organizational Psychology and Organizational Behavior 11, no. 1: 17–61. 10.1146/annurev-orgpsych-110721-040030.

[sjop70120-bib-0058] Primi, C. , K. Morsanyi , F. Chiesi , M. A. Donati , and J. Hamilton . 2016. “The Development and Testing of a New Version of the Cognitive Reflection Test Applying Item Response Theory (IRT).” Journal of Behavioral Decision Making 29, no. 5: 453–469. 10.1002/bdm.1883.

[sjop70120-bib-0059] Pytlik, N. , D. Soll , and S. Mehl . 2020. “Thinking Preferences and Conspiracy Belief: Intuitive Thinking and the Jumping to Conclusions‐Bias as a Basis for the Belief in Conspiracy Theories.” Frontiers in Psychiatry 11: 568942. 10.3389/fpsyt.2020.568942. PMC7530244 33061922

[sjop70120-bib-0060] Rizeq, J. , D. B. Flora , and M. E. Toplak . 2021. “An Examination of the Underlying Dimensional Structure of Three Domains of Contaminated Mindware: Paranormal Beliefs, Conspiracy Beliefs, and Anti‐Science Attitudes.” Thinking & Reasoning 27, no. 2: 187–211. 10.1080/13546783.2020.1759688.

[sjop70120-bib-0061] Rutjens, B. T. , and B. Većkalov . 2022. “Conspiracy Beliefs and Science Rejection.” Current Opinion in Psychology 46: 101392. 10.1016/j.copsyc.2022.101392. 35797743

[sjop70120-bib-0062] Sirota, M. , C. Dewberry , M. Juanchich , L. Valuš , and A. C. Marshall . 2021. “Measuring Cognitive Reflection Without Maths: Development and Validation of the Verbal Cognitive Reflection Test.” Journal of Behavioral Decision Making 34, no. 3: 322–343. 10.1002/bdm.2213.

[sjop70120-bib-0063] Šrol, J. 2022. “Individual Differences in Epistemically Suspect Beliefs: The Role of Analytic Thinking and Susceptibility to Cognitive Biases.” Thinking & Reasoning 28, no. 1: 125–162. 10.1080/13546783.2021.1938220.

[sjop70120-bib-0064] Ståhl, T. , and J. W. Van Prooijen . 2018. “Epistemic Rationality: Skepticism Toward Unfounded Beliefs Requires Sufficient Cognitive Ability and Motivation to Be Rational.” Personality and Individual Differences 122: 155–163. 10.1016/j.paid.2017.10.026.

[sjop70120-bib-0065] Stanley, M. L. , N. Barr , K. Peters , and P. Seli . 2021. “Analytic‐Thinking Predicts Hoax Beliefs and Helping Behaviors in Response to the COVID‐19 Pandemic.” Thinking & Reasoning 27, no. 3: 464–477. 10.1080/13546783.2020.1813806.

[sjop70120-bib-0066] Svedholm, A. M. , and M. Lindeman . 2013. “The Separate Roles of the Reflective Mind and Involuntary Inhibitory Control in Gatekeeping Paranormal Beliefs and the Underlying Intuitive Confusions.” British Journal of Psychology 104, no. 3: 303–319. 10.1111/j.2044-8295.2012.02118.x. 23848383

[sjop70120-bib-0067] Swami, V. , and D. Barron . 2021. “Rational Thinking Style, Rejection of Coronavirus (COVID‐ 19) Conspiracy Theories/Theorists, and Compliance With Mandated Requirements: Direct and Indirect Relationships in a Nationally Representative Sample of Adults From the United Kingdom.” Journal of Pacific Rim Psychology 15: 18344909211037385. 10.1177/18344909211037385.

[sjop70120-bib-0068] Swami, V. , M. Voracek , S. Stieger , U. S. Tran , and A. Furnham . 2014. “Analytic Thinking Reduces Belief in Conspiracy Theories.” Cognition 133, no. 3: 572–585. 10.1016/j.cognition.2014.08.006. 25217762

[sjop70120-bib-0069] Teovanović, P. , P. Lukić , Z. Zupan , A. Lazić , M. Ninković , and I. Žeželj . 2021. “Irrational Beliefs Differentially Predict Adherence to Guidelines and Pseudoscientific Practices During the COVID‐19 Pandemic.” Applied Cognitive Psychology 35, no. 2: 486–496. 10.1002/acp.3770. PMC7753549 33362344

[sjop70120-bib-0070] Thomson, K. S. , and D. M. Oppenheimer . 2016. “Investigating an Alternate Form of the Cognitive Reflection Test.” Judgment and Decision Making 11, no. 1: 99–113. 10.1017/S1930297500007622.

[sjop70120-bib-0071] Toplak, M. E. , R. F. West , and K. E. Stanovich . 2014. “Assessing Miserly Information Processing: An Expansion of the Cognitive Reflection Test.” Thinking & Reasoning 20, no. 2: 147–168. 10.1080/13546783.2013.844729.

[sjop70120-bib-0072] Uscinski, J. E. , K. Douglas , and S. Lewandowsky . 2017. “Climate Change Conspiracy Theories.” In Oxford Research Encyclopedia of Climate Science. 10.1093/acrefore/9780190228620.013.328.

[sjop70120-bib-0073] Uscinski, J. E. , and J. M. Parent . 2014. American Conspiracy Theories. Oxford University Press.

[sjop70120-bib-0074] Van Mulukom, V. , L. J. Pummerer , S. Alper , et al. 2022. “Antecedents and Consequences of COVID‐19 Conspiracy Beliefs: A Systematic Review.” Social Science & Medicine 301: 114912. 10.1016/j.socscimed.2022.114912. PMC8920084 35354105

[sjop70120-bib-0075] van Prooijen, J. W. 2017. “Why Education Predicts Decreased Belief in Conspiracy Theories.” Applied Cognitive Psychology 31, no. 1: 50–58. 10.1002/acp.3301. PMC5248629 28163371

[sjop70120-bib-0076] van Prooijen, J. W. , and R. Imhoff . 2022. “The Psychological Study of Conspiracy Theories: Strengths and Limitations.” Current Opinion in Psychology 48: 1–4. 10.1016/j.copsyc.2022.101465. 36191463

[sjop70120-bib-0077] Van Prooijen, J. W. , O. Klein , and J. Milošević Đorđević . 2020. “Social‐Cognitive Processes Underlying Belief in Conspiracy Theories.” In Routledge Handbook of Conspiracy Theories, edited by M. Butter and P. Knight , 168–180. Routledge.

[sjop70120-bib-0078] Washington, H. , and J. Cook . 2011. Climate Change Denial: Heads in the Sand. Routledge.

[sjop70120-bib-0079] World Medical Association . 2024. World Medical Association Declaration of Helsinki:Ethical Principles for Medical Research Involving Human Subjects. Accessed September 26, 2025. https://www.wma.net/policies‐post/wma‐declaration‐of‐helsinki/.

[sjop70120-bib-0080] Yelbuz, B. E. , E. Madan , and S. Alper . 2022. “Reflective Thinking Predicts Lower Conspiracy Beliefs: A Meta‐Analysis.” Judgment and Decision Making 17, no. 4: 720–744. 10.1017/S1930297500008913.

