# Supplementary material for: Latent Profile Analysis of Generic Conspiracy Beliefs‐ Differences in Science Acceptance, Analytical and Intuitive Thinking
Source: Scand J Psychol. 2026 May 22;67(6):1392–406. doi: 10.1111/sjop.70120 (PMC13739007; doi:10.1111/sjop.70120)
Supplement: Supplementary file 1 [file SJOP-67-1392-s001.docx]

Supplementary table S5 Acceptance of Climate and Vaccination science items Swedish translation

Climate science (R=reverse coded)

1. Jag anser att klimatet alltid förändras och vad vi ser just nu är bara naturliga variationer. (R)

2. Jag anser att större delen av uppvärmningen de senaste 50 åren beror på en ökning i koncentrationen av växthusgaser.

3. Jag anser att förbränningen av fossila bränslen de senaste 50 åren har orsakat allvarlig skada på planetens klimat.

4. Människans koldioxidutsläpp orsaker klimatförändring.

5. Människor är för obetydliga för att ha en märkbar inverkan på global temperatur. (R)

Vaccination science (R=reverse coded)

1. Jag anser att vaccin är ett säkert och tillförlitligt sätt att förhindra spridningen av sjukdomar som kan förebyggas.

2. Jag anser att vaccin har negativa sidoeffekter som är större än fördelarna med vaccination av barn. (R)

3. Vaccin är grundligt testade i laboratorium och skulle inte vara tillgängliga för allmänheten såvida det inte var känt att de är säkra.

4. Risken att vaccin skadar och dödar barn är större än deras hälsofördelar. (R)

5. Vaccinering är en av de mest väsentliga bidragen till folkhälsa.
