# Supplementary material for: Latent Profile Analysis of Generic Conspiracy Beliefs‐ Differences in Science Acceptance, Analytical and Intuitive Thinking
Source: Scand J Psychol. 2026 May 22;67(6):1392–406. doi: 10.1111/sjop.70120 (PMC13739007; doi:10.1111/sjop.70120)
Supplement: Supplementary file 2 [file SJOP-67-1392-s002.docx]

Supplementary table S2. GCBS instructions and items Swedish translation

**Uppfattningar om världen.**

Det debatteras ofta huruvida allmänheten verkligen informeras om hela sanningen vad gäller olika viktiga frågor. Den här korta undersökningen är utformad för att utvärdera dina uppfattningar vad gäller några av dessa ämnesområden. Vänligen använd följande skala för att besvara de här frågorna: Stämmer inte alls; Stämmer förmodligen inte; Osäker; Stämmer förmodligen; Stämmer helt

1. Staten och myndigheter är i hemlighet involverade i mord på oskyldiga medborgare och/eller välkända offentliga personer.

2. Makten hos världens ledare är underordnad den makt som innehas av en liten okänd grupp som är den som verkligen kontrollerar världspolitikens händelser.

3. Hemliga organisationer kommunicerar med utomjordiska varelser, men detta hemlighålls för allmänheten.

4. Vissa virus och/eller andra sjukdomar sprids medvetet men i hemlighet av någon organisation.

5. Forskargrupper manipulerar, fabricerar, eller undanhåller bevis för att vilseleda allmänheten.

6. Staten och myndigheter tillåter eller begår själva terroristhandlingar i sitt eget land, men döljer sin egen inblandning.

7. Viktiga världsomfattande beslut, som till exempel att gå i krig, fattas av en liten hemlig grupp människor.

8. Bevis för kontakt med utomjordingar undanhålls för allmänheten.

9. Teknologi som kan styra människors tankar används på människor utan att de vet om det.

10. Ny och avancerad teknologi som kan ge negativa konsekvenser för den rådande industrin undanhålls.

11. Staten och myndigheter använder människor som syndabockar för att dölja sin egen inblandning i kriminella aktiviteter.

12. Vissa viktiga internationella händelser är resultatet av aktiviteter utförda av en liten grupp människor som i hemlighet manipulerar världshändelser.

13. Vissa UFO observationer och rykten är planerade och iscensatta för att avleda allmänheten från verkliga kontakter med utomjordingar.

14. Experiment som innefattar nya läkemedel eller teknologier testas rutinmässigt på allmänheten utan dess kännedom eller medgivande.

15. En stor mängd viktig information hemlighålls medvetet från allmänheten på grund av egenintresse.
