# Supplementary material for: Latent Profile Analysis of Generic Conspiracy Beliefs‐ Differences in Science Acceptance, Analytical and Intuitive Thinking
Source: Scand J Psychol. 2026 May 22;67(6):1392–406. doi: 10.1111/sjop.70120 (PMC13739007; doi:10.1111/sjop.70120)
Supplement: Supplementary file 3 [file SJOP-67-1392-s003.docx]

Supplementary table S4 Acceptance of Climate and Vaccination science items original version

Climate science (R=reverse coded)

1. I believe that the climate is always changing and what we are currently observing is just natural fluctuation. (R)

2. I believe that most of the warming over the last 50 years is due to the increase in greenhouse gas concentrations.

3. I believe that the burning of fossil fuels over the last 50 years has caused serious damage to the planet’s climate.

4. Human CO2 emissions cause climate change.

5. Humans are too insignificant to have an appreciable impact on global temperature. (R)

Vaccination science (R=reverse coded)

1. I believe that vaccines are a safe and reliable way to help avert the spread of preventable diseases.

2. I believe that vaccines have negative side effects that outweigh the benefits of vaccination for children. (R)

3. Vaccines are thoroughly tested in the laboratory and wouldn’t be made available to the public unless it was known that they are safe.

4. The risk of vaccinations to maim and kill children outweighs their health benefits. (R)

5. Vaccinations are one of the most significant contributions to public health.
