# Supplementary material for: Latent Profile Analysis of Generic Conspiracy Beliefs‐ Differences in Science Acceptance, Analytical and Intuitive Thinking
Source: Scand J Psychol. 2026 May 22;67(6):1392–406. doi: 10.1111/sjop.70120 (PMC13739007; doi:10.1111/sjop.70120)
Supplement: Supplementary file 4 [file SJOP-67-1392-s004.docx]

Supplementary table S1. GCBS instructions and items original version

**Beliefs About the World**

**There is often debate about whether or not the public is told the whole truth about various important issues. This brief survey is designed to assess your beliefs about some of these subjects. Please indicate the degree to which you believe each statement is likely to be true on the following scale: Definitely not true; Probably not true; Not sure/cannot decide; Probably true; Definitely true**

1. The government is involved in the murder of innocent citizens and/or well-known public figures, and keeps this a secret

2. The power held by heads of state is second to that of small unknown groups who really control world politics

3. Secret organizations communicate with extraterrestrials, but keep this fact from the public

4. The spread of certain viruses and/or diseases is the result of the deliberate, concealed efforts of some organization

5. Groups of scientists manipulate, fabricate, or suppress evidence in order to deceive the public

6. The government permits or perpetrates acts of terrorism on its own soil, disguising its involvement

7. A small, secret group of people is responsible for making all major world decisions, such as going to war

8. Evidence of alien contact is being concealed from the public

9. Technology with mind-control capacities is used on people without their knowledge

10. New and advanced technology which would harm current industry is being suppressed

11. The government uses people as patsies to hide its involvement in criminal activity

12. Certain significant events have been the result of the activity of a small group who secretly manipulate world events

13. Some UFO sightings and rumors are planned or staged in order to distract the public from real alien contact

14. Experiments involving new drugs or technologies are routinely carried out on the public without their knowledge or consent

15. A lot of important information is deliberately concealed from the public out of self-interest
