# Supplementary material for: Latent Profile Analysis of Generic Conspiracy Beliefs‐ Differences in Science Acceptance, Analytical and Intuitive Thinking
Source: Scand J Psychol. 2026 May 22;67(6):1392–406. doi: 10.1111/sjop.70120 (PMC13739007; doi:10.1111/sjop.70120)
Supplement: Supplementary file 5 [file SJOP-67-1392-s005.docx]

Supplementary table S3 CRT items original version and Swedish translation

Original version

1. If it takes 5 machines 5 minutes to make 5 widgets, how long would it take 100 machines to make 100 widgets?” (correct answer= 5 minutes and intuitive answer= 100 minutes)
2. In a lake, there is a patch of lily pads. Every day, the patch doubles in size. If it takes 48 days for the patch to cover the entire lake, how long would it take for the patch to cover half of the lake?” (correct answer= 47 days, intuitive answer= 24)
3. In an athletics team, tall members are three times more likely to win a medal than short members. This year the team has won 60 medals so far. How many of these have been won by short athletes?“ (correct answer= 15 medals, intuitive answer= 20 medals)
4. Simon decided to invest $8,000 in the stock market one day early in 2008. Six months after he invested, on July 17, the stocks he had purchased were down 50%. Fortunately for Simon, from July 17 to October 17, the stocks he had purchased went up 75%. At this point, Simon has:
A. broken even in the stock market,
B. is ahead of where he began,
C. has lost money.

(correct answer= c, intuitive answer= b)

Swedish translation

1. Om det tar 5 maskiner 5 minuter att tillverka 5 ”manicker”, hur lång tid tar det då för 100 maskiner att tillverka 100 ”manicker”? ________ minuter.
2. På en sjö växer det näckrosor. Varje dag så fördubblas ytan som näckrosorna täcker. Om det tar 48 dagar för näckrosorna att täcka hela sjön, hur många dagar tar det för näckrosorna att täcka halva sjön? __________ dagar.
3. I ett friidrottslag har långa deltagare tre gånger större chans att vinna medalj än korta deltagare. Det här året har laget än så länge vunnit 60 medaljer. Hur många av dessa har vunnits av korta friidrottare? ______ medaljer
4. En dag i början av 2008 beslutade sig Simon för att investera 8 000 kr på aktiemarknaden. Sex månader efter hans investering, den 17:e juli, hade aktierna han köpte gått ner med 50%. Lyckligtvis så gick aktierna han köpte upp med 75% från den 17:e juli till den 17:e oktober. Vid denna tidpunkt hade Simon
A. gått jämnt ut på aktiemarknaden.
B. mer pengar än då han började investera.
C. förlorat pengar.
